# Unilateral external ophthalmoplegia in Miller Fisher syndrome: case report

**DOI:** 10.1186/1471-2415-7-7

**Published:** 2007-04-17

**Authors:** Jonathan Smith, Lucy Clarke, Philip Severn, Robert Boyce

**Affiliations:** 1 Sunderland Eye Infirmary, Sunderland, Queen Alexandra Road, Sunderland, SR2 9HP, UK

## Abstract

**Background:**

A description of the diagnostic features of Miller Fisher syndrome.

**Case presentation:**

The clinical presentation, investigation, and subsequent progress of our patient with clinical unilateral external ophthalmoplegia.

**Conclusion:**

Our case demonstrates the presentation of clinical unilateral external ophthalmoplegia as part of the full triad of Miller Fisher syndrome.

## Background

Miller Fisher syndrome was originally described in 1956 as a triad of total external ophthalmoplegia, ataxia and loss of tendon reflexes [1]. Since the original description acute phase immunoglobulin G (IgG) antibodies to GQ1b ganglioside have been recognised as a serum marker for Miller Fisher syndrome [2]. Anti GQ1b IgG antibodies are found in over 90% of cases of Miller Fisher syndrome and are highly disease specific [3]. We report a case of a 32-year old gentleman with clinical unilateral external ophthalmoplegia in Miller Fisher syndrome confirmed by positive GQ1b antibody titre.

## Case presentation

A 32-year old gentleman presented to the ophthalmic accident and emergency department with a four-day history of worsening horizontal and vertical diplopia. He denied any other ophthalmic or neurological symptoms at presentation. Other than a recent mild coryzal illness he was fit and well. He had no significant past ocular or medical history and took no regular medications.

**Figure 1:**
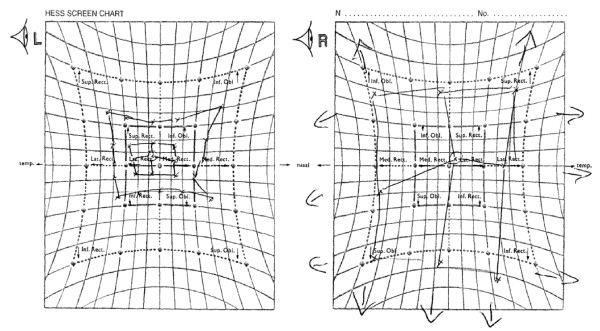
Hess chart recorded at presentation.

Ocular examination within the accident and emergency department showed a one-millimetre ptosis of the left upper lid, with limitation of left ocular movements in all directions of gaze. Right ocular movements were full. The remainder of the ocular and cranial nerve examination was normal. Eye movement testing showed a -2/-3 reduction in left ocular movements in all directions of gaze, with an associated +2 over-action in the corresponding right ocular movements. Figure 1 shows the Hess chart recorded at presentation.

A CT scan of his head with contrast showed no abnormality and the patient was transferred to the regional neurosciences centre for further investigation where the patient was found to be ataxic on heel to toe walk and the deep tendon reflexes were absent. Further investigation including an MRI, serology, lumbar puncture and Tensilon test were normal. Anti GQ1b IgG antibodies were positive with a significant titre by laboratory reference range, confirming the clinical diagnosis of Miller Fisher syndrome. With conservative management the patient's signs and symptoms resolved spontaneously within five weeks of presentation, leaving no permanent sequelae.

## Conclusion

The marked asymmetry of the ophthalmoplegia in this case is unusual, and to our knowledge only a single previous report of a clinical unilateral external ophthalmoplegia as part of the full triad of Miller Fisher syndrome has been documented within the literature [4]. Acute partial bilateral and unilateral ophthalmoparesis has been documented in association with anti GQ1b IgG antibodies, however these patients were not ataxic and the presence of deep tendon reflexes were variable [5], or neurological examination was normal [6].

This case demonstrates that uniocular external ophthalmoplegia can occur as part of the full triad in Miller Fisher syndrome. This reinforces the importance of considering systemic autoimmune diseases normally associated with bilateral ophthalmoparesis, in the differential diagnosis of a patient presenting with apparently unilateral signs.

## Competing interests

The author(s) declare that they have no competing interests.

## Authors' contributions

JS and LC drafted and referenced the case study.

PS and RB revised the manuscript.

All authors read and approved the final manuscript.

## Pre-publication history

The pre-publication history for this paper can be accessed here:


